# Epidemiology and microbiology of fracture-related infection: a multicenter study in Northeast China

**DOI:** 10.1186/s13018-021-02629-6

**Published:** 2021-08-12

**Authors:** Baisheng Wang, Xiaoguang Xiao, Jingdong Zhang, Wenfeng Han, Salad Abdirahman Hersi, Xin Tang

**Affiliations:** 1 Department of Orthopedics, First Affiliated Hospital of Dalian Medical University, Dalian, Liaoning 116011 People’s Republic of China; 2 Dalian Medical University, Dalian, Liaoning 116044 People’s Republic of China; 3 Department of Clinical Laboratory, First Affiliated Hospital of Dalian Medical University, Dalian, Liaoning, 116011 People’s Republic of China; 4 Department of Orthopedics, Northeast International Hospital, Shenyang, Liaoning 110004 People’s Republic of China; 5 Department of Orthopedics, The General Hospital of Northern Theater Command, Shenyang, Liaoning 110016 People’s Republic of China

**Keywords:** Epidemiology, Microbiology, Fracture-related infection, Bacterial resistance

## Abstract

**Objective:**

This study aimed to explore the epidemiological and microbiological characteristics of fracture-related infection (FRI), analyze the drug resistance characteristics of major pathogens, and provide timely and relatively complete clinical and microbiological data for antimicrobial treatment of FRI.

**Methods:**

The clinical and microbiological data of patients with FRI from January 1, 2011, to December 31, 2020, were collected from three tertiary hospitals in Northeast China. The automatic microbial analysis system was used for strain identification and drug susceptibility testing, and the drug susceptibility results were determined in accordance with the latest Clinical and Laboratory Standards Institute (CLSI) criteria (as applicable each year).

**Results:**

A total of 744 patients with FRI were enrolled. The incidence of FRI was about 1.5%, and 81.7% were male patients, with an average age of 48.98 ± 16.01 years. Open fractures accounted for 64.8%. Motor crush (32.8%) and falling (29.8%) were the main causes of injuries. The common sites of infection were the tibia and fibula (47.6%), femur (11.8%), foot (11.8%), and hand (11.6%). A total of 566 pathogenic bacteria were cultured in 378 patients with positive bacterial cultures, of which 53.0% were Gram-positive bacteria and 47.0% were Gram-negative bacteria. The most common pathogen at all sites of infection is *Staphylococcus aureus*. *Staphylococcus aureus* had a high resistance rate to penicillin (PEN), erythromycin (ERY), and clindamycin (CLI), exceeding 50%. Methicillin-resistant *Staphylococcus aureus* (MRSA) was more than 80% resistant to CLI and ERY.

**Conclusions:**

The incidence of FRI in Northeast China was at a low level among major medical centers nationwide. *Staphylococcus aureus* was still the main pathogen causing bone infections, and the proportion of MRSA was lower than reported abroad, but we have observed an increase in the proportion of infections. *Enterobacteriaceae* have a higher resistance rate to third-generation cephalosporins and quinolones. For *Enterobacteriaceae*, other sensitive treatment drugs should be selected clinically.

## Introduction

In recent years, with the rapid development of China’s economy, the number of patients with open injury and multiple fractures caused by road and industrial accidents has increased dramatically [1]. Open and multiple injuries often cause infection. Infection is one of the common serious complications in orthopedics. Long treatment cycles, high treatment costs, and poor prognosis bring huge physical and mental harm to patients, as well as huge challenges to doctors and healthcare systems. The total medical cost of infected patients after tibial fracture is 6.5 times that of uninfected patients, antibiotic treatment time is 11 times that of uninfected patients, and the hospital stay is 7.7 times that of uninfected patients [2]. The risk of fracture-related infection (FRI) depends on the location of the injury, severity, and the accompanying injury and physiological state of the host. The incidence of infection after closed low-energy fractures is about 1%, and the incidence of infection after complex open fractures is about 15% [3, 4]. FRI is usually caused by exogenous factors such as initial trauma or surgery [5].

The clinical features of FRI are diverse and may be affected by geographic location, climate, time, and injury factors [6–8]. The epidemiological and microbiological characteristics of patients with FRI are particularly important for guiding clinical treatment. The current lack of epidemiological and microbiological research reports on FRI. To solve this problem, we retrospectively analyzed 744 patients with FRI from 2011 to 2020 in three large tertiary hospitals in Northeast China. This study explores the epidemiological and microbiological characteristics of patients with FRI and analyzes the characteristics of drug-resistant bacteria spectrum of main pathogens. This study can provide evidence-based medicine for the empirical treatment of FRI with antibiotics.

## Methods

This study reviewed the medical records of patients hospitalized for fractures from January 1, 2011, to December 31, 2020, and selected cases of patients with FRI that met the inclusion criteria, including gender, age, injury factors, infection site, comorbidities, bacterial culture, and drug sensitivity test results. The diagnostic criteria for FRI referred to the diagnostic criteria by the Association for the Study of Internal Fixation (AO/ASIF) [9]. Microscan WalkAway 96 plus (Beckman, USA) or VITEK 2 Compact (Meyer, France) automatic microbial analysis system is used for strain identification and antimicrobial susceptibility testing. The results of antimicrobial susceptibility testing were determined in accordance with the latest CLSI criteria (as applicable each year). The minimum inhibitory concentrations (MICs) were used to detect methicillin-resistant *S. aureus*. Quality control strains include *S. aureus* (ATCC 25923), *E. coli* (ATCC25922), and *P. aeruginosa* (ATCC27853) (National Health Commission Clinical Laboratory Center).

### Inclusion criteria

1. Patient eligible for diagnosis of FRI

### Exclusion criteria

1. Incomplete medical record
2. Multi-site infection
3. Pathological fracture such as bone tuberculosis and bone tumor
4. Periprosthetic infection
5. Infections of the skull, sternum, and ribs

### Diagnostic criteria for FRI

The diagnostic criteria of FRI are divided into confirmatory criteria (the present infection can be determined as long as a confirmatory criterion is met) and suggestive criteria (features of FRI that related to infection but need to be further investigated). There are four confirmatory criteria: (1) fistula, sinus, or wound breakdown (with communication to the bone or the implant); (2) purulent drainage from the wound or the presence of pus during surgery; (3) phenotypically indistinguishable pathogens identified by culture from at least two separate deep tissue/implant specimens taken during an operative intervention; and (4) presence of microorganisms in deep tissue taken during an operative intervention, as confirmed by histopathological examination using specific staining techniques for bacteria or fungi. Suggestive diagnosis criteria include clinical signs, inflammatory signs, radiological signs, new-onset joint effusion, persistent wound drainage, and pathogenic organism culture results. The detailed diagnostic criteria of FRI refer to the consensus by the Association for the Study of Internal Fixation (AO/ASIF) [9].

### Ethics approval and informed consent

The collected data was anonymized and de-identified before data analysis. The Institutional Review Board of First Affiliated Hospital of Dalian Medical University granted a waiver of written informed consent and provided authorization for this study (number PJ-KS-KY-2021-31). The study protocol was registered with the Chinese Clinical Trial Registry. All related procedures were performed in accordance with relevant guidelines and regulations.

### Statistics analysis

Statistical analysis was performed with the SPSS 22.0 software (SPSS Inc., Chicago, IL, USA). Application of WHONET 5.6 software was performed for bacterial resistance analysis. Statistics was described as mean ± standard deviation (SD) or as the count and percentage as appropriate. A chi-square test or Kruskal–Wallis *H* test was used to analyze the difference in count data. All the significance tests were two-sided tests, and *P* < 0.05 was considered statistically significant.

## Results

**Fig. 1:**
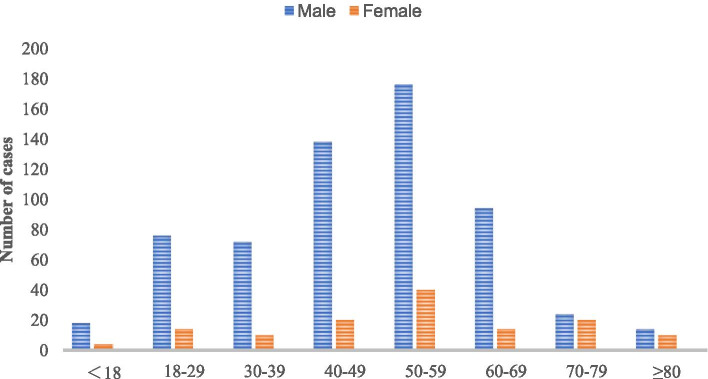
Distribution of age and gender

**Fig. 2:**
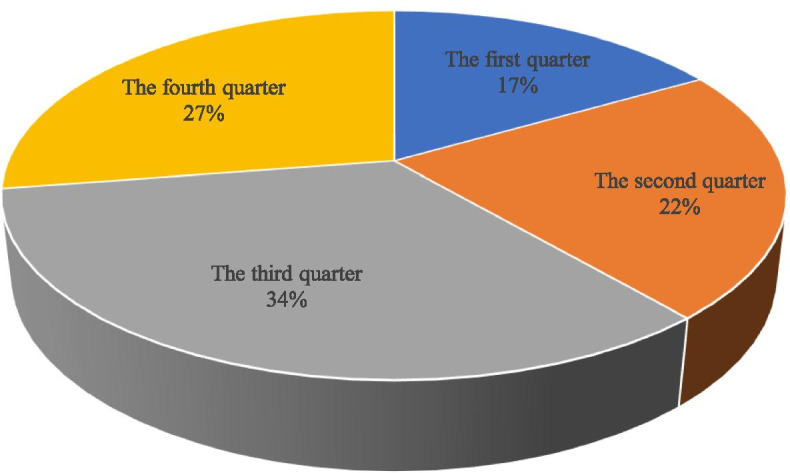
Seasonal distribution of cases

**Fig. 3:**
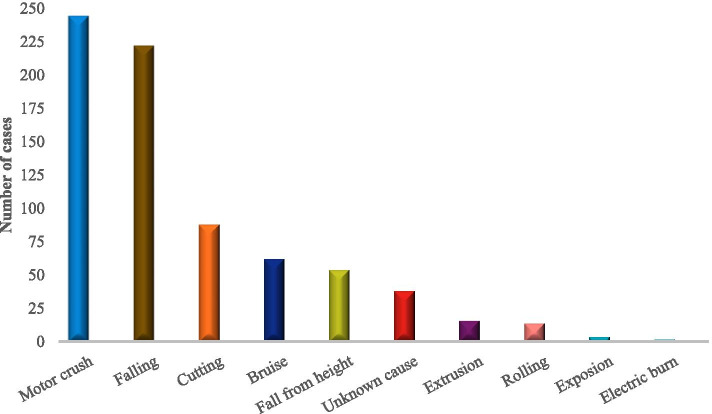
Distribution of injury mechanism

**Fig. 4:**
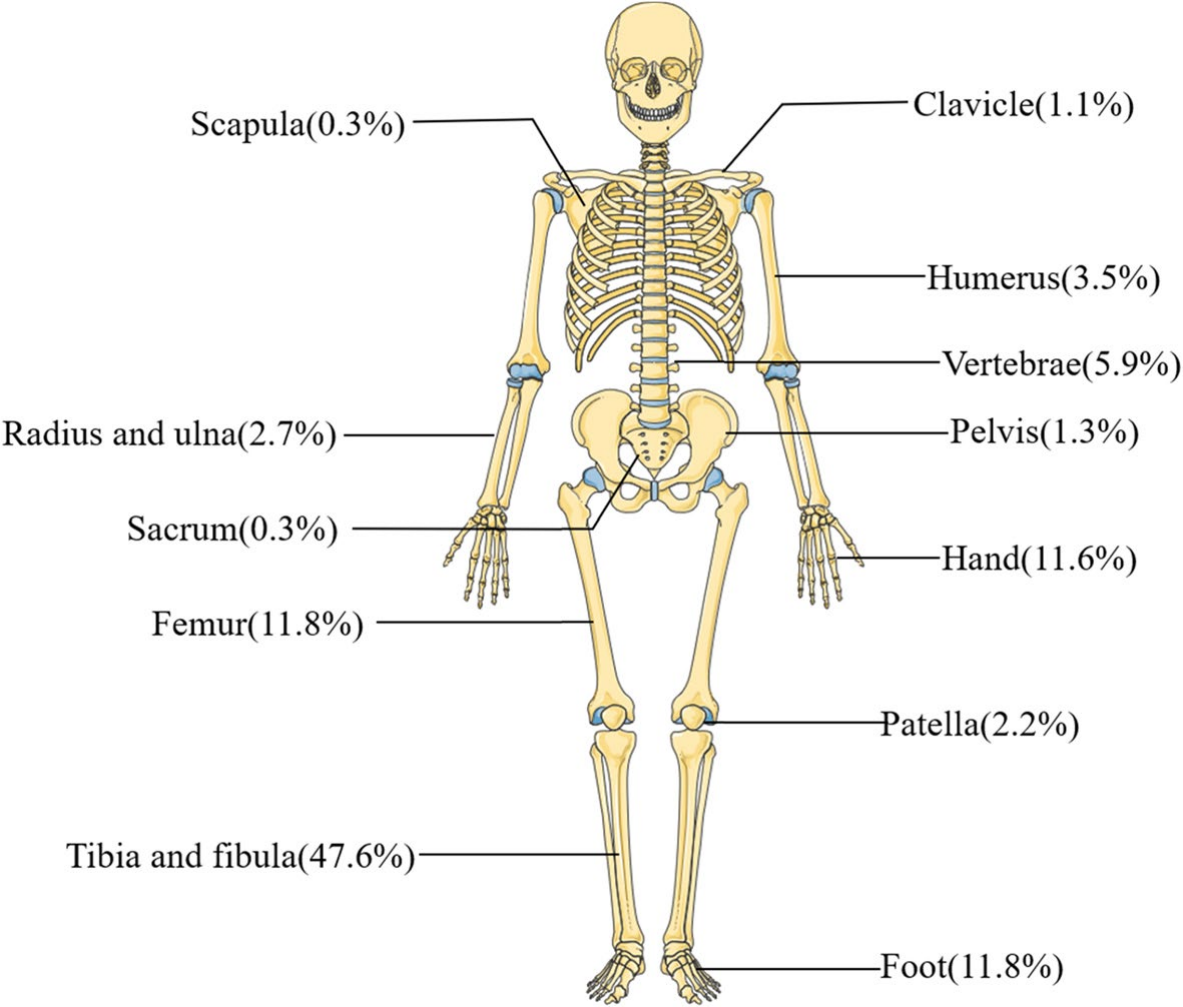
Distribution of infection sites

According to the inclusion and exclusion criteria, 744 patients with FRI from 2011 to 2020 were screened out from 48,186 fracture patients who were treated surgically in our centers. The incidence of FRI was about 1.5%, of which 378 (50.8%) patients had positive bacterial cultures. The study included 608 males (81.7%) and 136 females (18.3%) with an average age of 48.98 ± 16.01 years. The most patients were 50 to 59 (216 cases, 29.03%). Four hundred fifty-two (60.8%) patients came from the outpatient department, and 292 (39.2%) patients came from the emergency department. There were 482 (64.8%) open fractures and 504 (67.7%) single fractures. One hundred twelve (15.1%) patients had neurovascular injury, 68 (9.1%) patients had diabetes, and 70 (9.4%) patients had hypertension (Table 1 and Fig. 1). The largest number of patients was in the third quarter, 33.9% of the year (Fig. 2). The most common causes of injuries were motor crush (244 cases, 32.8%) and falling (222 cases, 29.8%) (Fig. 3). There were 142 (19.1%) cases of upper extremity bone infection and 556 (74.7%) of lower extremity bone infection, and the common sites of infection were the tibia and fibula (354 cases, 47.6%), femur (88 cases, 11.8%), foot (88 cases, 11.8%), and hand (86 cases, 11.6%) (Fig. 4).

**Table 1:** Demographic characteristics of 744 patients with fracture-related infection (2011–2020)

	2011–2012	2013–2014	2015–2016	2017–2018	2019–2020	Total
Age^a^	47 ± 16	49 ± 17	50 ± 15	50 ± 16	48 ± 15	49 ± 16
Male/female	104/22	112/20	146/26	140/32	106/36	608/136
Admission(*n* [%])						
Outpatient	78 (62)	86 (65)	112 (65)	104 (60)	72 (51)	452 (61)
Emergency	48 (38)	46 (35)	60 (35)	68 (40)	70 (49)	292 (39)
Fracture type (*n* [%])						
Single fracture	88 (70)	86 (65)	118 (69)	118 (69)	94 (66)	504 (68)
Multiple fracture	38 (30)	46 (35)	54 (31)	54 (31)	48 (34)	240 (32)
Wound type (*n* [%])						
Open injury	84 (67)	96 (73)	108 (63)	110 (64)	84 (59)	482 (65)
Closed injury	42 (33)	36 (27)	64 (37)	62 (36)	58 (41)	262 (35)
Infection site (*n* [%])						
Upper limb	16 (13)	26 (21)	34 (20)	46 (27)	20 (14)	142 (19)
Lower limb	100 (79)	104 (78)	122 (71)	116 (67)	114 (80)	556 (75)
Vertebrae and sacrococcyx	10 (8)	2 (1)	16 (9)	10 (6)	8 (6)	46 (6)
Side (*n* [%])						
Left	66 (52)	66 (50)	78 (45)	80 (47)	70 (49)	360 (48)
Axis	10 (8)	2 (2)	18 (10)	8 (5)	12 (8)	50 (7)
Right	50 (40)	64 (49)	76 (44)	84 (49)	60 (42)	334 (45)
Injury mechanism (*n* [%])						
Motor crush	38 (30)	50 (38)	62 (36)	44 (26)	50 (35)	244 (33)
Falling	38 (30)	42 (32)	52 (30)	56 (33)	34 (24)	222 (30)
Fall from height	14 (11)	8 (6)	14 (8)	8 (5)	10 (7)	54 (7)
Bruise	12 (10)	6 (5)	14 (8)	18 (10)	12 (8)	62 (8)
Cutting	12 (10)	16 (12)	22 (13)	26 (15)	12 (8)	88 (12)
Others	12 (10)	10 (8)	8 (5)	20 (12)	24 (17)	74 (10)
Complication (*n* [%])						
Diabetes	12 (10)	10 (8)	12 (7)	26 (15)	8 (6)	68 (9)
Hypertension	4 (3)	8 (6)	22 (13)	28 (16)	8 (6)	70 (9)
Neurovascular injury	26 (21)	16 (12)	20 (12)	34 (20)	16 (11)	112 (15)
Compartment syndrome	4 (3)	2 (2)	6 (3)	6 (3)	2 (1)	20 (3)
Cardiovascular disease	4 (3)	0 (0)	10 (6)	8 (5)	2 (1)	24 (3)
Shock	10 (8)	4 (3)	10 (6)	8 (5)	14 (10)	46 (6)
Chest and abdomen injuries	4 (3)	6 (5)	8 (5)	10 (6)	6 (4)	34 (5)
Brain injury	2 (2)	6 (5)	8 (5)	4 (2)	0 (0)	20 (3)

^a^The values are given as the mean and the standard deviation

A total of 566 pathogenic bacteria were cultured from 378 patients with positive bacterial culture, including 300 (53.0%) Gram-positive bacteria and 266 (47.0%) Gram-negative bacteria. The common pathogens are *Staphylococcus aureus* (166 strains, 29.3%), *Staphylococcus epidermidis* (44 strains, 7.8%), *Pseudomonas aeruginosa* (42 strains, 7.4%), *Escherichia coli* (40 strains, 7.1%), *Klebsiella pneumoniae* (36 strains, 6.4%), and *Enterobacter cloacae* (28 strains, 4.9%). MRSA in 2020 (12 strains, 37.5%) increased compared with 2011 (4 strains, 14.3%), but the difference was not statistically significant (*P* > 0.05) (Table 2).

**Table 2:** Main pathogens of FRI (2011–2020)

	2011–2012	2013–2014	2015–2016	2017–2018	2019–2020	Total
SAU	28	34	36	36	32	166
MSSA	24	28	26	26	20	124
MRSA^a^	4	6	10	10	12	42
SEP	16	2	10	8	8	44
PAE	8	10	8	14	2	42
ECO	12	2	6	12	8	40
KPN	6	0	6	16	8	36
Total	70	48	66	86	58	328

^a^Comparison between the proportion of MRSA in 2019–2020 and 2011–2012, *P* > 0.05

**Fig. 5:**
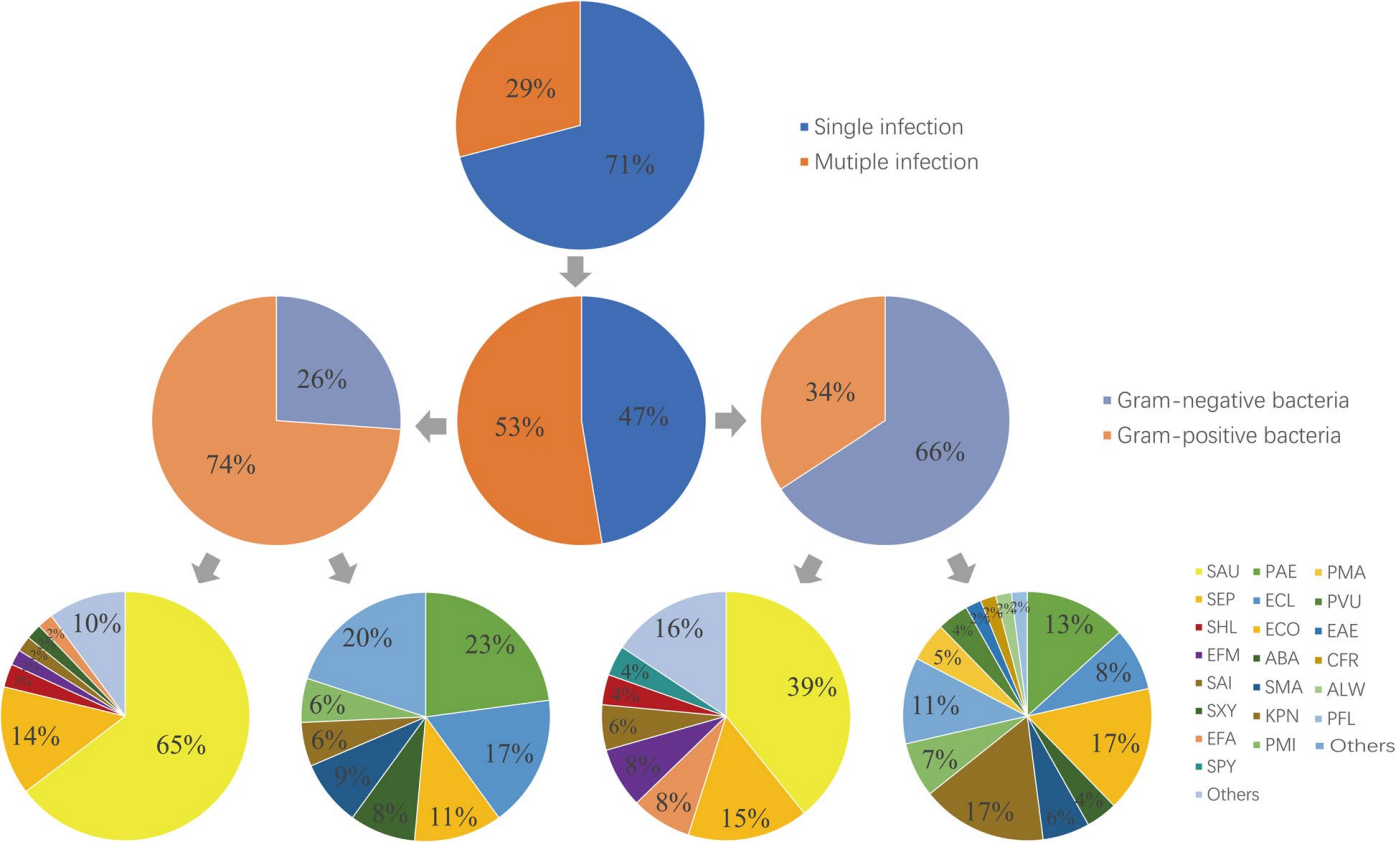
Distribution of pathogenic bacteria with FRI (2011–2020)

**Fig. 6:**
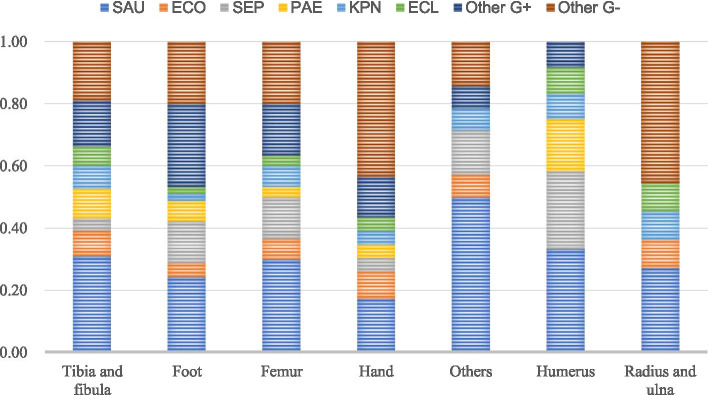
Distribution of pathogenic bacteria at the site of infection

Among the 378 patients with positive bacterial culture, 268 (70.9%) were infected by a single pathogen and 110 (29.1%) were infected by multiple pathogens. Among the patients with single pathogen infection, there were 198 (73.9%) strains of Gram-positive bacteria and 70 (26.1%) strains of Gram-negative bacteria. The main Gram-positive bacteria were *S. aureus* (128,64.6%) and *S. epidermidis* (28, 14.1%). The main Gram-negative bacteria were *P. aeruginosa* (16 strains, 22.9%), *E. cloacae* (12, 17.1%), and *E. coli* (8 strains, 11.4%). Among the patients with multiple infections, 102 (34.2%) were Gram-positive bacteria and 196 (65.8%) were Gram-negative bacteria. The main Gram-positive bacteria were *S. aureus* (40, 39.2%) and *S. epidermidis* (16, 15.7%). The main Gram-negative bacteria were *E. coli* (32, 16.3%), *P. aeruginosa* (32, 16.3%), and *K. pneumoniae* (26, 13.3%). *S. aureus* was the most common pathogenic bacteria in all infection sites, and the hand and forearm had a higher infection rate of *Gram-negative bacteria* (Figs. 5 and 6).

*S. aureus* had high resistance rates to penicillin (PEN), erythromycin (ERY), and clindamycin (CLI), all exceeding 50%. MRSA was completely resistant to PEN, oxacillin (OXA), amoxicillin/clavulanic acid (AMC), and ceftriaxone (CRO). The resistance rate of MRSA to CLI and ERY was more than 80%. The resistance rate of *S. epidermidis* to PEN, OXA, AMC, CRO, and ERY was more than 80% (Tables 3 and 4). The resistance rate of *E. coli* to ampicillin (AMP), cefazolin (CZO), and cefuroxime (CXM) was more than 70%, and it was completely sensitive to carbapenemases such as imipenem (IPM), meropenem (MEM), and ertapenem (ETP). The resistance rate of *K. pneumoniae* to ampicillin (ATM), ciprofloxacin (CIP), CZO, CXM, CRO, and compound sulfamethoxazole (SXT) was more than 50%. The resistance rate of *P. aeruginosa* to commonly used drugs was less than 30% (Table 5).

**Table 3:** Antimicrobial resistance of the main Gram-positive bacteria

Antimicrobial	*S. aureus* (*n* = 166)			*S. epidermidis* (*n* = 44)		
	*R* (%)	*I* (%)	*S* (%)	*R* (%)	*I* (%)	*S* (%)
PEN	96.4	0	3.6	100.0	0	0
OXA	25.3	0	74.7	81.8	0	18.2
AMC	25.3	0	74.7	86.4	0	13.6
CRO	25.3	0	74.7	86.4	0	13.6
GEN	24.1	1.2	74.7	36.4	4.5	59.1
RIF	3.6	0	96.4	13.6	0	86.4
CIP	15.7	18.1	66.3	59.1	0	40.9
LVX	14.5	1.2	84.3	59.1	0	40.9
MFX	14.5	1.2	84.3	40.9	18.2	40.9
SXT	4.8	0	95.2	40.9	0	59.1
CLI	51.8	2.4	45.8	59.1	0	40.9
DAP	0	0	100.0	0	0	100.0
ERY	69.9	2.4	27.7	81.8	0	18.2
LNZ	0	0	100.0	0	0	100.0
VAN	0	0	100.0	0	0	100.0
QDA	3.6	0	96.4	4.5	0	95.5
TCY	15.7	3.6	80.7	22.7	4.5	72.7

**Table 4:** Antimicrobial resistance of MRSA and MSSA

Antimicrobial	MRSA (*n* = 42)			MSSA (*n* = 124)		
	*R* (%)	*I* (%)	*S* (%)	*R* (%)	*I* (%)	*S* (%)
PEN	100.0	0	0	95.2	0	4.8
OXA	100.0	0	0	0	0	100.0
AMC	100.0	0	0	0	0	100.0
CRO	100.0	0	0	0	00	100.0
GEN	33.3	4.8	61.9	21.0	0	79.0
RIF	14.3	0	85.7	0	0	100.0
CIP	19.0	9.5	71.4	14.5	21.0	64.5
LVX	19.0	4.8	76.2	12.9	0	87.1
MFX	19.0	0	81.0	12.9	1.6	85.5
SXT	14.3	0	85.7	1.6	0	98.4
CLI	81.0	0	19.0	41.9	3.2	54.9
DAP	0	0	100.0	0	0	100.0
ERY	85.7	4.8	9.5	64.5	1.6	33.9
LNZ	0	0	100.0	0	0	100.0
VAN	0	0	100.0	0	0	100.0
QDA	14.3	0	85.7	0	0	100.0
TCY	38.1	4.8	57.1	8.1	3.2	88.7

**Table 5:** Antimicrobial resistance of the main Gram-negative bacteria

Antimicrobial	*E. coli* (*n* = 40)			*K. pneumoniae* (*n* = 36)			*P. aeruginosa* (*n* = 42)		
	*R* (%)	*I* (%)	*S* (%)	*R* (%)	*I* (%)	*S* (%)	*R* (%)	*I* (%)	*S* (%)
CSL	20.0	15.0	65.0	16.7	16.7	66.6	4.8	23.8	71.4
TZP	15.0	5.0	80.0	16.7	5.6	77.8	9.5	9.5	81.0
CAZ	45.0	5.0	50.0	33.3	16.7	50.0	9.5	9.5	81.0
FEP	50.0	0	50.0	44.4	5.6	50	14.3	4.8	80.9
ATM	50.0	0	50.0	61.1	0	38.9	23.8	14.3	61.9
IPM	0	0	100.0	16.7	0	83.3	9.5	0	90.5
MEM	0	0	100.0	16.7	0	83.3	9.5	0	90.5
AMK	15.0	5.0	80.0	5.6	0	94.4	0	9.5	90.5
GEN	55.0	0	45.0	22.2	5.6	72.2	14.3	0	85.7
TOB	40.0	15.0	45.0	27.8	16.7	55.6	9.5	0	90.5
CIP	65.0	0	35.0	50.0	0	50.0	0	9.5	90.5
LVX	55.0	5.0	40.0	38.9	11.1	50.0	0	9.5	90.5
AMP	95.0	0	5.0	100.0	0	0	–	–	–
CZO	75.0	0	25.0	55.6	0	44.4	–	–	–
CXM	65.0	5.0	30.0	55.6	0	44.4	–	–	–
CRO	60.0	0	40.0	55.6	0	44.4	–	–	–
FOX	25.0	5.0	70.0	38.9	0	61.1	–	–	–
ETP	0	0	100.0	16.7	0	83.3	–	–	–
SXT	65.0	0	35.0	55.6	0	44.4	–	–	–

## Discussion

FRI is one of the most daunting and challenging complications in the treatment of trauma patients, which can lead to delayed healing, permanent loss of function, and even amputation [10, 11]. FRI can also cause huge socio-economic cost and lead to a significantly prolonged recovery period for patients. Antibiotics play an important role in the prevention and treatment of FRI [12]. The treatment of FRI is complicated and requires a standardized, long-term antibiotic treatment regimen [13]. Clarifying the clinical characteristics and dominant pathogenic strains of current FRI is of great significance for guiding clinical treatment. There have been no reports of epidemiological studies on FRI in Northeast China, in order to clarify the epidemiological and microbiological characteristics of FRI in Northeast China to guide the use of clinical empirical antibiotics. We conducted a retrospective study on patients with FRI from 2011 to 2020 in three tertiary hospitals in Northeast China.

The incidence of FRI in our research centers for 10 years is 1.5%, which is at a low level compared to other research (0.4 to 16.1%) [14]. This may be related to our strict screening criteria. First of all, previous studies mostly used SSI defined by the CDC as the diagnostic criteria of infection [15–20]. Compared with the diagnostic criteria of SSI, the diagnostic criteria recommended by FRI consensus are more detailed, which exclude the patients with superficial SSI infection [9]. Secondly, previous studies have mostly focused on a specific part. These parts have a high risk of postoperative infection, such as the tibia, ankle joint, and calcaneus [16, 17, 21, 22]. Finally, due to the large number of variables in the clinical and microbial characteristics of the patients, we excluded medical records with missing data. These may be the reason why the incidence rate of infection in our study is lower than other studies. The third quarter is a period of high incidence of FRI, and there is a significant time difference in the risk of infection. The mechanism is not clear, and related studies speculate that the risk of infection may be related to climate, temperature, and humidity [23, 24]. Motor crush is the most common cause of injury (32.8%), which is inseparable from the rapid development of traffic development in China. Among patients with FRI, men accounted for approximately 81.8%, which is similar to the Kremers report [8]. The report believes that men are more likely to engage in heavy manual labor or high-risk activities, and the increase in road and industrial accidents has led to more male patients. In this study, the 50–59 years old accounted for the highest proportion, probably because this age group is engaged in high-risk operations, which may easily lead to fractures, and this part of the population often has diseases that increase the risk of infection, such as diabetes [25]. Diabetes is a common complication of FRI in this study. Immunity is an important factor in the occurrence and transformation of osteomyelitis [26]. Older people have weaker immunity and probably more prone to infection [27]. The most common infection sites were the tibia and fibula (47.6%), femur (11.8%), foot bone (11.8%), and hand bone (11.6%), which is similar to related reports [28, 29]. There is less soft tissue around the tibia, and the lack of blood supply after trauma or surgery increases the chance of wound infection.

Bacterial culture results showed Gram-positive bacteria (53.0%) and Gram-negative bacteria (47.0%). As expected, *S. aureus* (29.3%) is the most common pathogenic bacteria, of which 25.3% are MRSA. Gram-negative bacteria are mainly *P. aeruginosa* (7.4%), *E. coli* (7.1%), and *K. pneumoniae* (6.4%), which are similar to related research [7, 30]. *Enterobacteriaceae* accounted for 29.3% in this study, which is similar to a recent study that reported that 35.5% of bone infections were related to *Enterobacteriaceae*. The higher incidence of *Enterobacteriaceae* infection may be related to the fact that most of these patients suffered open fracture after direct trauma, and/or infection caused by soft tissue injury [28]. *P. aeruginosa* is common in our study, accounting for 12.8%, which is quite worrying because *P. aeruginosa* has been found to be associated with an increased risk of recurrence of osteomyelitis [31, 32]. 25.3% of *S. aureus* are resistant to oxacillin in our study, which is lower than reports in Brazil (35.5%) and the Middle East (60.5%) [32, 33]. This may be related to more cautious use of antibiotics and more strict control of nosocomial infection in these centers. We recommend that if FRI is suspected, antibiotics should not be used before surgical debridement unless the patient has sepsis [34]. During the operation, sterile instruments were used to collect multiple tissue samples for microbiological and histopathological examinations. If signs of FRI (e.g., pus) are found during surgery, empirical intravenous antibacterial therapy should be started immediately after sampling. The empirical antimicrobial treatment should be continued until the microbiological results are available, and then reassessment. Our empirical antibiotic strategy is third-generation cephalosporin combined with vancomycin and then adjusted according to the culture results [13]. The resistance rate of *S. aureus* to rifampicin and fluoroquinolone is less than 20%, and it is recommended as the first-line oral treatment for *Staphylococcus* [35]. When *S. aureus* is resistant to oxacillin, intravenous vancomycin is recommended first. Coagulase-negative *Staphylococcus* (e.g., *S. epidermidis*) is resistant to oxacillin, which is consistent with the treatment strategy for MRSA. *Enterobacteriaceae* have a high resistance rate to third-generation cephalosporins, and we speculate that their resistance is usually related to the production of extended-spectrum β-lactamase (ESBLs) [36]. However, this study lacks relevant experimental data and cannot determine its resistance mechanism. Fluoroquinolone is the cornerstone for the treatment of bone and joint infections caused by Gram-negative bacteria [37]. But the resistance rate of *Enterobacteriaceae* to fluoroquinolones in this study is higher than 50%. According to the results of the *Enterobacteriaceae* susceptibility test in our study, we recommend the use of carbapenem antibiotics (e.g., meropenem and imipenem). Although *P. aeruginosa* has a high sensitivity to fluoroquinolones in this study, we do not recommend fluoroquinolones as the initial treatment after debridement because they may have a higher resistance rate. We recommend third-generation cephalosporins or carbapenems as the initial treatment for non-fermenting bacteria (e.g., *P. aeruginosa*) [13]. Carbapenem-resistant strains are rare in this study. However, due to the global spread of carbapenemase-resistant *Enterobacteriaceae* bacteria since 2012, it is expected that the resistance will increase in the future, requiring to be paid more attention by clinicians [38].

This study includes several limitations. This is a retrospective study with a relatively small sample size. We did not keep fresh samples for complete gene sequencing to understand gene mutations and protein expression levels. China has a vast territory; ethnic groups, climates, living customs, and eating habits of different regions are diverse. Our results only represent cases of FRI in this region. The types of antibiotics selected by medical institutions in different periods are different. Although we have selected as many commonly used antibiotics as possible in the research design to obtain the results of bacterial drug sensitivity tests, it still cannot fully reflect the actual situation. Our research included the period of the Sars-COVID-19 pandemic, and we do not know any influences on the epidemiology of FRI yet. So, it would be a known bias.

## Conclusion

The incidence of FRI in our centers was at a low level in large medical centers across the country. *Staphylococcus aureus* was still the main pathogen causing bone infections. The proportion of MRSA was still lower than reported abroad, but we have observed an increase in the proportion of infections. *Enterobacteriaceae* had higher resistance rates to third-generation cephalosporins and fluoroquinolones. Other sensitive treatment drugs should be selected clinically for *Enterobacteriaceae*. This study showed the epidemiological, clinical, and microbiological characteristics of FRI in three centers. These results can provide a basis for formulating effective preventive measures and treatment plans and reduce the burden of treatment of FRI.

## Data Availability

All data generated or analyzed during this study are included in this published article.

## Abbreviations

FRI: Fracture-related infection
CLSI: Clinical and Laboratory Standards Institute
MRSA: Methicillin-resistant *Staphylococcus aureus*
ESBL: Extended-spectrum β-lactamase
AO/ASIF: Association for the Study of Internal Fixation
MICs: Minimum inhibitory concentrations
